# 聚多巴胺纳米纤维膜固相萃取-超高效液相色谱-串联质谱检测淡水鱼中四环素类和氟喹诺酮类药物残留

**DOI:** 10.3724/SP.J.1123.2020.12026

**Published:** 2021-06-08

**Authors:** Sihui LIANG, Hairong DAI, Huayin ZHANG, Jian LI, Qiuping ZHANG, Qian XU, Chunmin WANG

**Affiliations:** 1.东南大学公共卫生学院营养与食品卫生学系, 江苏 南京 210009; 1. Department of Nutrition and Food Hygiene, School of Public Health, Southeast University, Nanjing 210009, China; 2.苏州市疾病预防控制中心, 江苏 苏州 215100; 2. Suzhou Center for Disease Control and Prevention, Suzhou 215100, China; 3.东南大学环境与医学工程教育部重点实验室, 江苏 南京 210009; 3. Key Laboratory of Ministry of Education of Environment and Medical Engineering, Southeast University, Nanjing 210009, China

**Keywords:** 超高效液相色谱-串联质谱, 固相萃取, 聚多巴胺, 纳米纤维膜, 药物残留, 淡水鱼, ultra performance liquid chromatography-tandem mass spectrometry (UPLC-MS/MS), solid phase extraction (SPE), polyaniline (PDA), nanofiber mat (NFsM), drug residues, fish

## Abstract

制备聚多巴胺(PDA)修饰的聚苯乙烯纳米纤维膜(PS NFsM)作为固相萃取吸附介质,可快速提取淡水鱼中3种四环素类(四环素、金霉素、土霉素)和3种氟喹诺酮类(恩诺沙星、环丙沙星、诺氟沙星)药物残留,结合超高效液相色谱-串联质谱(UPLC-MS/MS),建立了药物残留检测的新方法。利用静电纺丝法制备了聚苯乙烯纳米纤维膜,将其作为模板,通过自聚合作用,进行聚多巴胺功能化修饰,得到PDA-PS NFsM材料。对制得的PS NFsM和PDA-PS NFsM材料进行傅里叶红外光谱和场发射扫描电镜表征,证明PDA的成功修饰,修饰后的纳米纤维表面粗糙,呈现核-壳形貌,纤维内部为蜂窝状多孔结构。以空白加标样品的回收率为指标,对PDA-PS NFsM材料的用量、离子强度、样品溶液的流速、洗脱液和突破体积等影响SPE的因素进行考察及条件优化,确定了最佳的SPE条件。该方法对6种目标物的检出限为0.3~1.5 μg/kg,定量限为1.0~5.0 μg/kg,低于国家标准和行业标准;在各目标物的线性范围内均有良好的线性关系,决定系数(*R*^2^)大于0.999,方法的回收率为94.37%~102.82%,日间和日内的相对标准偏差(RSD)均小于10%,与国家标准和行业标准相当。通过固相萃取前后的基质效应对比,表明PDA-PS NFsM具有优秀的净化能力。最后,通过实际样品分析验证了方法的实际应用可行性。该文建立的基于PDA-PS NFsM材料的SPE方法是一种高效环保的方法,可为淡水鱼中药物残留的常规监测提供技术支持。

## Abstract (en)

Tetracyclines and fluoroquinolones are common antibacterial drugs used in aquaculture, and their residues may pose a risk to human health. The low concentration of drug residues and complex matrixes such as fats and proteins in aquatic products necessitate the urgent development of efficient sample pretreatment methods. Solid phase extraction (SPE) is the most common sample pretreatment method, in which the core is an adsorbent. Compared with traditional SPE adsorbents, nanofiber mat (NFsM) has more interaction sites because of their large specific surface area. Furthermore, NFsMs modified with specific functional groups can significantly improve the extraction efficiency of tetracyclines and fluoroquinolones. Polydopamine (PDA) is spontaneously synthesized by the oxidative self-polymerization of dopamine-hydrochloride in alkaline solutions (pH>7.5). Because of its rich amino and catechol groups, PDA can form *π-π* stacking, electrostatic attraction, hydrophobic interaction, and hydrogen bonding interactions with target molecules. By exploiting the above advantages, polystyrene (PS) NFsM, as a template, was prepared by the electrostatic spinning method, and PDA-PS NFsM was obtained by functional modification of PDA through self-polymerization. Fourier transform infrared spectroscopy (FT-IR) and field-emission scanning electron microscopy (FESEM) were used to characterize the synthesized PS NFsM and PDA-PS NFsM. It was proved that PDA was successfully modified on the PS NFsM, with the SEM images revealing a rough outer core shell structure and an inner honeycomb structure. Subsequently, the handmade SPE column with PDA-PS NFsM was completed. A novel and efficient screening analytical method based on PDA-PS NFsM for the simultaneous determination of three tetracyclines (tetracycline (TET), chlortetracycline (CTC), and oxytetracycline (OTC)) and three fluoroquinolones (enrofloxacin (ENR), ciprofloxacin (CIP), and norfloxacin (NOR)) in fish by ultra performance liquid chromatography-tandem mass spectrometry (UPLC-MS/MS) was established. The SPE procedure was optimized to develop an efficient method for sample preparation. Evaluate parameters including the amount of NFsM usage, ionic strength, flow rate of the sample solution, composition of eluent, and breakthrough volume were investigated. Only (20±0.1) mg of PDA-PS NFsM was sufficient to completely adsorb the targets, and the analytes retained on NFsM could be eluted by 1 mL of formic acid-ethyl acetate (containing 20% methanol) (1∶99, v/v). The residues were redissolved in 0.1 mL 10% methanol aqueous solution containing 0.2% formic acid. In addition, no adjustment of the pH and ionic strength of the sample solutions was required, and the breakthrough volume was 50 mL. The limits of detection (LODs) and limits of quantification (LOQs) of the six target compounds were measured at 3 times and 10 times the signal-to-noise ratio (*S/N*), respectively. The LODs and LOQs were 0.3-1.5 μg/kg and 1.0-5.0 μg/kg, respectively. The linear ranges of the six target compounds were LOQ-1000 μg/kg, and the coefficient of determination (*R*^2^) was greater than 0.999. To evaluate the accuracy and precision, blank spiked samples at three levels (low, medium, and high) were prepared for the recovery experiments, and each level with six parallel samples (*n*=6). The recoveries ranged from 94.37% to 102.82%, with intra-day and inter-day relative standard deviations of 2.38% to 8.06% and 4.10% to 9.10%, respectively. To evaluate the purification capacity of PDA-PS NFsM, the matrix effects before and after SPE were calculated and compared. Matrix effects before SPE were -12.98% to -38.68%. After the completion of SPEbased on PDA-PS NFsM, the matrix effect of each target analyte was significantly reduced to -2.15% to -7.36%, which proved the significant matrix removal capacity of PDA-PS NFsM. Finally, the practicality of this method was evaluated by using it to analyze real samples. This SPE method based on PDA-PS NFsM is efficient, practical, and environmentally friendly, and it has great potential for use in the routine monitoring of drug residues in fish.

水产品是优质蛋白质、多种不饱和脂肪酸等多种营养物质的主要食物来源,为满足人们的食用需求,多采用集约化养殖,这种高密度养殖也给防疫带来了压力^[1,2]^。四环素类和氟喹诺酮类药物是常用的抗菌类药物,在水产品中的残留检出率也较高,对消费者的健康有潜在风险^[3]^。因此,许多国家和组织,包括美国、中国、日本、欧盟(EU)等均建立了水产品等动物源性食品中药物的最大残留限量(MRL)^[4,5,6,7,8]^,作为食品安全监测工作的依据。

超高效液相色谱-串联质谱法(UPLC-MS/MS)是动物源性食品中药物残留检测的主要方法,但由于药物残留水平较低(μg/mL或μg/L),且实际样品中脂肪、蛋白质等常严重抑制质谱的电离效率,进而影响分析结果的准确度和灵敏度^[9,10]^,因此,UPLC-MS/MS检测前必须进行样品预处理,以去除杂质、富集目标物。在我国检测水产品中兽药残留的国家标准、行业标准^[11,12,13]^和相关的研究报道^[14,15,16]^中,固相萃取(SPE)是最常采用的样品前处理方法。SPE技术主要基于萃取介质对目标物的吸附作用,因此新型吸附介质的开发应用成为其重要的发展方向之一^[17,18,19,20]^。纳米纤维(nanofibers, NFs)是最典型的纳米材料之一,静电纺丝法(electrospinning,简称电纺)作为制备NFs的通用方法,为制得纳米纤维膜(NFsM)提供了技术支持^[21]^。根据目标物性质和检测样品的特点,NFsM可被功能化修饰,获得的功能化NFsM是一种新型高效的SPE介质^[22]^。笔者课题组自2008年起即开展了基于功能化NFsM的SPE工作^[23,24,25,26,27,28,29,30]^,研制了多种吸附和解吸附效率双高的功能化NFsM,并在食品安全分析、环境监测、生物样本分析等方面实现了良好应用。

聚多巴胺(PDA)的结构中含有儿茶酚和胺类官能团,是由盐酸多巴胺在弱碱性溶液(pH>7.5)中通过氧化自聚合而合成的^[31]^。PDA具有良好的亲水性和生物相容性,可与目标分析物形成各种相互作用,如*π-π*堆积、静电作用、疏水作用和氢键等^[32]^。因此,PDA被认为是的一种新型吸附介质^[33,34]^,已应用于SPE^[35]^、固相微萃取(SPME)^[36]^等样品前处理方法。PDA修饰后的NFsM是一种“强强联合”的高效SPE介质,其兼具丰富的吸附机制、快速的传质效能和高效的基质净化能力。多项研究提示^[36,37,38]^, PDA功能化修饰的纳米纤维有望成为一种优越的SPE吸附介质,但将其应用于动物源性食品中药物残留检测的研究还未见报道。

本文研制PDA功能化的聚苯乙烯(PS)纳米纤维膜(PDA-PS NFsM),建立了基于PDA-PS NFsM的新型SPE方法,联合UPLC-MS/MS,检测在水产品中检出率较高的3种四环素(四环素、土霉素、金霉素)和3种氟喹诺酮(恩诺沙星、环丙沙星、诺氟沙星),对方法的准确度和精密度等效能进行了评价,并通过对实际淡水鱼样品中6种目标物的检测,验证了方法的实际应用性。

## 1 实验部分

### 1.1 仪器与试剂

UPLC超高效液相色谱仪、Xevo TQD三重四极杆质谱仪(配TQD质量检测器、Acquity自动采样器、ESI源,美国Waters公司), Quanta 200 FEG场发射扫描电子显微镜(捷克FEI公司), TENSOR27傅里叶变换红外光谱仪(日本Shimadzu公司), 24孔固相萃取装置(美国Supelco公司), Heraeus Multifuge X1R型台式离心机(美国Thermo公司)。

聚苯乙烯(*M*_r_260000)、盐酸多巴胺(纯度为98%)和Tris缓冲溶液购于上海阿拉丁有限公司。乙腈和甲醇均为HPLC级,购自德国Merck公司。配制EDTA-McIlvaine’s缓冲液所用试剂十二水合磷酸氢二钠、一水合柠檬酸、乙二胺四乙酸,以及十二烷基苯磺酸钠、四氢呋喃和*N*,*N*-二甲基甲酰胺均购自上海国药化工公司。标准物质:四环素(TET)、金霉素(CTC)、土霉素(OTC)、恩诺沙星(ENR)、环丙沙星(CIP)、诺氟沙星(NOR),纯度均为98.0%,均购自美国Sigma公司。

准确称取10.0 mg各标准物质,分别溶解于10.0 mL甲醇,配制成质量浓度为1000 mg/L的标准储备溶液。分别取一定体积的储备溶液,混合,用甲醇稀释得各目标物质量浓度均为10 mg/L的混合标准溶液。用超纯水稀释混合标准溶液,得到所需浓度的标准工作溶液。所有的溶液均保存于4 ℃备用。

### 1.2 PDA-PS NFsM材料的制备

称取0.3 g十二烷基苯磺酸钠,溶于10 mL四氢呋喃中,再加入20 mL *N*,*N*-二甲基甲酰胺,混合均匀后,称取6 g聚苯乙烯粉末溶于上述溶液中,在磁力搅拌器上搅拌至均一溶液。用静电纺丝法制备纳米纤维膜,电压为19 kV,流速为1.0 mL/h,纺制时间为0.5 h,即可制得直径约为(12±1) cm、厚度约为(100±10) μm近似圆形的PS NFsM材料。将铝箔纸接收板在室温干燥2 h后揭下,作为基底膜进行聚多巴胺修饰。

称取2 g盐酸多巴胺,溶于200 mL Tris缓冲液(10 mmol/L, pH 8.5)中,将PS NFsM平铺于大玻璃平皿中,用上述配制完成的盐酸多巴胺溶液将其完全浸润,保鲜膜密封后,于60 ℃避光水浴反应12 h后,用超纯水清洗至浸出液澄清,烘干,得到厚度约为190 μm的PDA-PS NFsM材料。

将如上制得的PDA-PS NFsM材料用打孔器裁剪,得到质量为(20.0±0.1) mg、直径为1 cm的PDA-PS NFsM圆片,然后夹在两个筛板之间,放入内径为1 cm的洁净空柱管底部,即得自制SPE小柱,如图1所示。此SPE柱依次用0.5 mL去离子水、0.5 mL甲酸-乙酸乙酯(含20%甲醇)(1∶99, v/v)和0.5 mL去离子水活化后备用。

**图 1:**
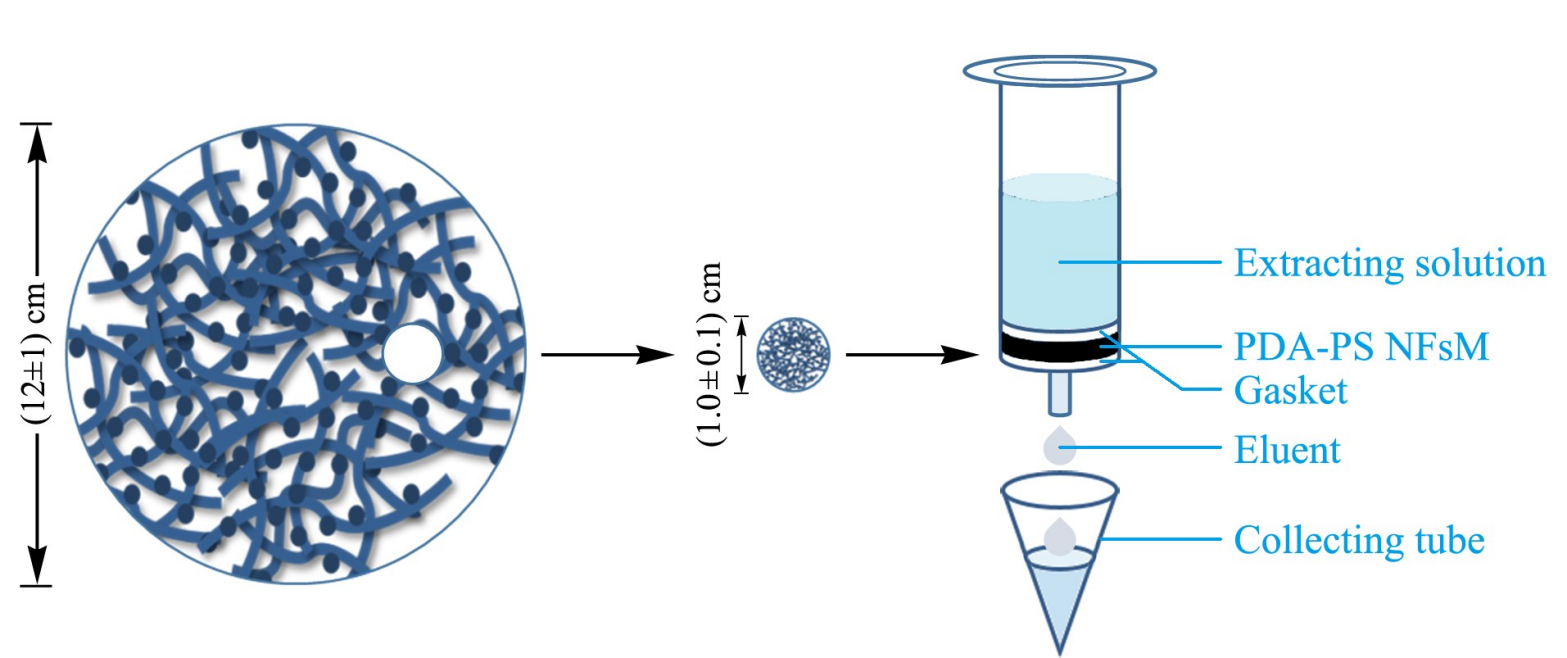
自制SPE小柱示意图

### 1.3 样品前处理

在苏州市姑苏区农贸市场采集淡水鱼样品共16份。每份淡水鱼样品分别取其身体两面的背部、腹部、尾部鱼肉各约5 g,混合后绞碎成肉糜,置于50 mL离心管中,密塞并准确标记样品号,于-20 ℃保存,一周内完成检测。

称取(2.00±0.05) g室温下解冻后的鱼肉糜,置于15 mL具塞离心管中,加入2.00 mL提取液(乙腈-EDTA-McIlvaine’s缓冲液(1∶1, v/v)),以2000 r/min涡旋混合5 min,于4 ℃以11000 r/min离心5 min。取1 mL上清液,用超纯水稀释至10 mL,得到样品溶液。将10 mL样品溶液以3 mL/min的速率通过1.2节所述的SPE小柱,用1 mL甲酸-乙酸乙酯(含20%甲醇)(1∶99, v/v)直接洗脱,洗脱液氮吹至干,用0.1 mL 10%甲醇水溶液(含0.2%甲酸)复溶后,进行UPLC-MS/MS检测。

### 1.4 分析条件

1.4.1 色谱条件

色谱柱:Acquity UPLC BEH C18柱(50 mm×2.1 mm, 1.7 μm);柱温:40 ℃;流动相:A为甲醇,B为0.1%甲酸溶液;流速:0.2 mL/min。线性洗脱程序:0~3.0 min, 95%A; 3.0~4.8 min, 95%A~5%A; 4.8~5.0 min, 5%A~95%A; 5.0~5.5 min, 95%A。进样量:5 μL。

1.4.2 质谱参数

离子源:ESI源;扫描模式:正离子扫描模式;监测方式:多反应监测(MRM)模式;毛细管电压:3.0 kV,脱溶剂温度:350 ℃,脱溶剂气流速:800 L/h。各目标物的具体质谱参数见表1。

**表 1:** 6种目标物的质谱参数

Compound	t_R_/min	Precursor ion (m/z)	Product ions (m/z)	Collision energies/V	Cone voltage/V
Norfloxacin (NOR)	1.68	320.2	302.2^*^, 276.2, 233.2	21, 16, 25	31
Ciprofloxacin (CIP)	1.72	332.2	288.2^*^, 314.2	18, 19	31
Oxytetracycline (OTC)	1.74	461.3	426.3^*^, 443.3, 201.2	19, 13, 37	26
Enrofloxacin (ENR)	1.78	360.3	316.3^*^, 245.2, 342.1	18, 27, 23	41
Tetracycline (TET)	1.83	445.3	410.2^*^, 154.1	18, 27	27
Chlortetracycline (CTC)	2.03	479.3	462.2^*^, 444.2, 154.1	18, 22, 29	27

^*^ Quantitative ion.

## 2 结果与讨论

### 2.1 PDA-PS NFsM材料的表征

对制得的PS NFsM和PDA-PS NFsM材料进行了傅里叶红外光谱和场发射扫描电镜表征。如图2所示,在1287、1488、1615和3200 cm^-1^处出现了强的吸收峰,分别对应氨基上N-H的振动吸收峰、芳香环的特征峰、C=C的特征峰和邻苯二酚上-OH的吸收峰,说明PDA-PS NFsM材料制备成功。PS NFsM表面光滑,平均直径为700~900 nm(见图3a)。PDA包覆于PS NFsM形成了核-壳形貌的PDA-PS NFsM(见图3b),其表面较PS NFsM明显粗糙,纤维直径增大,内部呈蜂窝状多孔结构。

**图 2:**
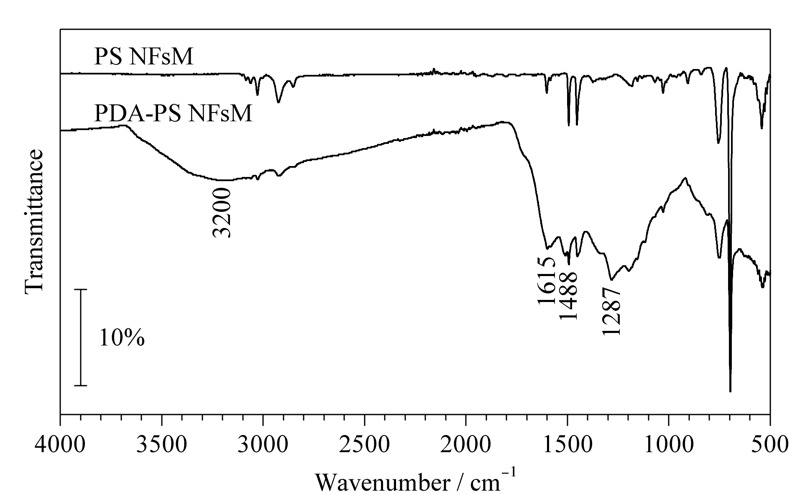
PS NFsM和PDA-PS NFsM的傅里叶红外光谱图

**图 3:**
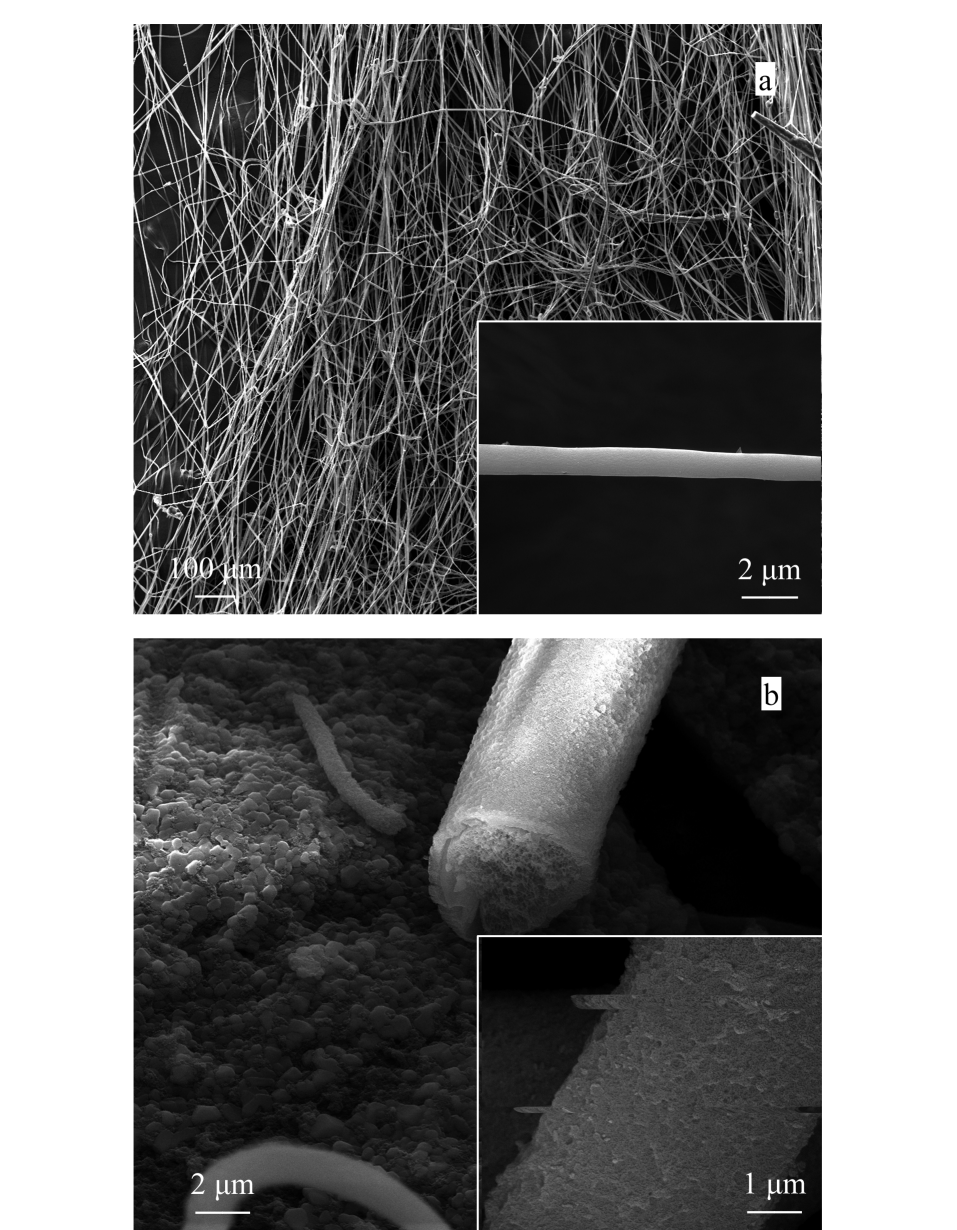
(a)PS NFsM和(b)PDA-PS NFsM的场发射扫描电镜图

### 2.2 SPE影响因素的考察及条件优化

PDA-PS NFsM材料的用量、离子强度、样品溶液的流速和洗脱液等,都会对萃取效率产生影响。以所有目标物均未检出的淡水鱼样品作为空白样品,按1.3节进行预处理,得到空白样品溶液,在其中加入一定体积的混合标准溶液,以回收率为指标,采用样品基质匹配的加标溶液进行SPE影响因素的考察及条件优化。

2.2.1 吸附剂的使用量

考察自制SPE小柱中PDA-PS NFsM材料的质量对6种目标化合物萃取效率的影响。在对基底膜进行PDA修饰时,改变60 ℃避光水浴的反应时间(3、6、9、12和15 h),即可得到厚度约为130、150、170、190、210 μm的PDA-PS NFsM材料,然后使用内径为1 cm的打孔器获得质量分别为(5.0±0.1)、(15.0±0.1)、(20.0±0.1)和(25.0±0.1) mg的PDA-PS NFsM圆片。从图4a可以看出,采用5~20 mg PDA-PS NFsM材料时,回收率呈上升趋势,采用20~25 mg时,回收率趋于平稳,说明20 mg 的PDA-PS NFsM材料足够用于吸附目标分析物。

**图 4:**
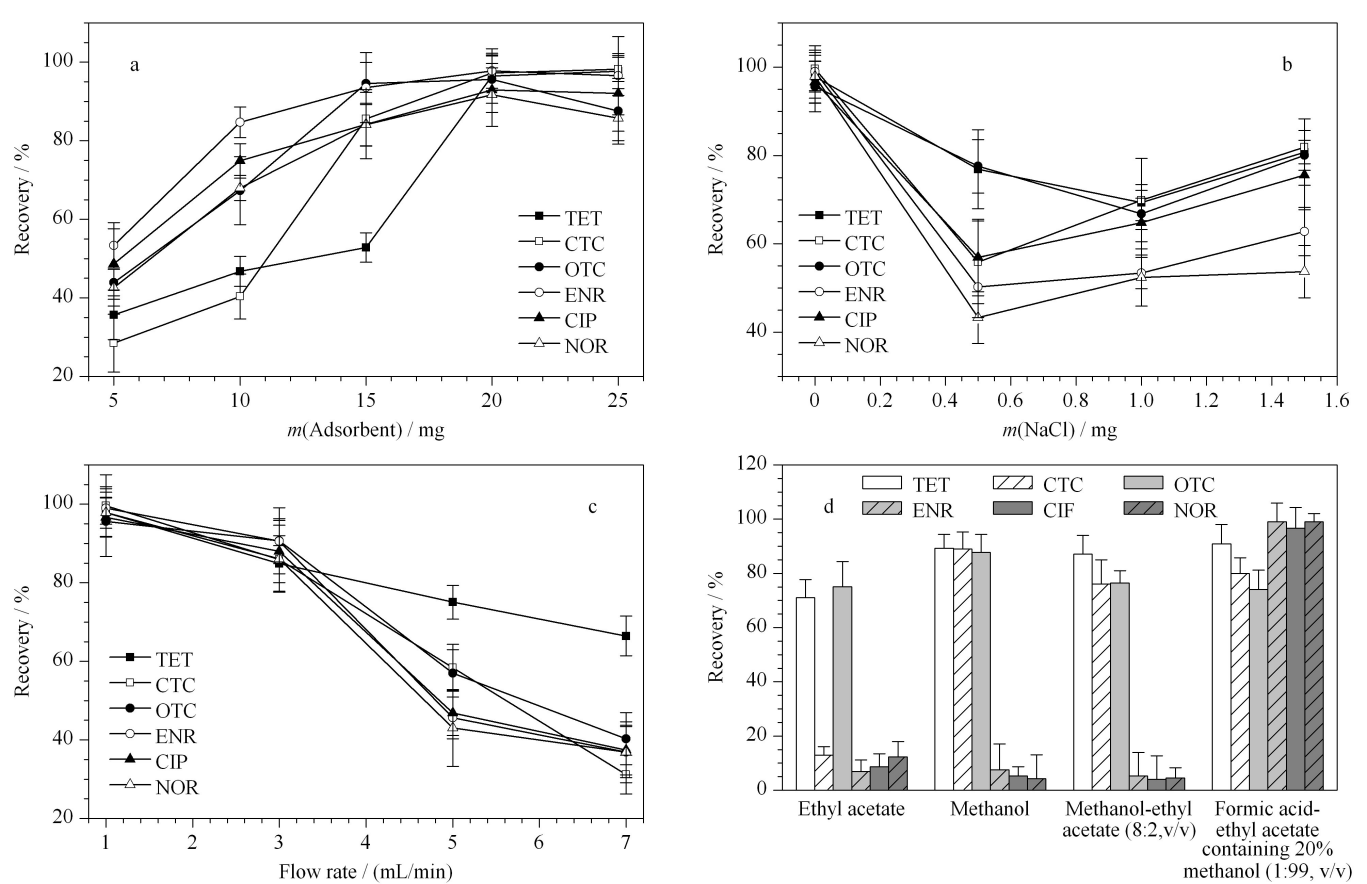
(a)吸附剂的使用量、(b)离子强度、(c)样品溶液的流速和(d)洗脱液对6种目标化合物萃取效率的影响(*n*=4)

2.2.2 离子强度

用含有0~1.5 g NaCl的样品溶液考察离子强度对SPE萃取效率的影响。在10 mL的样品溶液中分别加入0、0.5、1.0、1.5 g NaCl,然后进行固相萃取,检测洗脱液中6种目标物的含量。从图4b可以看出,当NaCl的添加量由0 g增加至0.5 g时,6种目标物的回收率均下降;当NaCl的添加量为0.5~1.0 g时,TET和OTC的回收率仍下降,而其他4种目标物的回收率小幅回升,这可能是由于各目标物对离子强度的敏感性不同;当NaCl的添加量为1.0~1.5 g时,6种目标物的回收率均小幅上升,但仍低于NaCl添加量为0 g时。因此不需要在样品溶液中添加NaCl。

2.2.3 样品溶液的流速

在保证回收率的前提下,提高流速可以缩短前处理的时间。样品溶液分别以1~7 mL/min的流速通过装填有20 mg PDA-PS NFsM材料的SPE小柱,考察流速对6种目标物回收率的影响。如图4c所示,当流速从1 mL/min增加至3 mL/min时,6种目标物的回收率虽有所下降,但基本处于90%以上;当流速大于3 mL/min时,6种目标物的回收率均大幅度下降。当流速从1 mL/min增加至3 mL/min,上样时间可从10 min缩短至3.3 min。因此经过综合考虑,选择3 mL/min作为样品溶液的流速。

2.2.4 洗脱液组成

选择洗脱液的标准是能够尽可能多地将吸附于PDA-PS NFsM材料的目标分析物解吸。研究了弱极性溶剂乙酸乙酯,强极性溶剂甲醇、甲醇-乙酸乙酯(8∶2, v/v)和甲酸-乙酸乙酯(含20%甲醇)(1∶99, v/v)对6种目标物的洗脱能力。结果表明,氟喹诺酮类药物对pH值敏感,在酸性条件下的加标回收率显著提高。综合考虑后,当使用甲酸-乙酸乙酯(含20%甲醇)(1∶99, v/v)时,6种目标物的回收率最好(见图4d)。

### 2.3 突破体积的考察

在固相萃取时,随样品溶液的加入,吸附介质对目标物吸附逐渐达到饱和,当目标物不再被吸附时所能流过的最大样品溶液体积即为穿透体积。当样品溶液体积超过突破体积时,萃取效率会显著降低。因此,本文对突破体积进行了考察,结果如图5所示,当样品溶液体积为10~50 mL时,各目标物回收率均>93%,当样品溶液体积增大至60~70 mL时,回收率有明显的下降。因此样品的突破体积约为50 mL。本文采用10 mL的样品溶液,并未超过突破体积,不会影响萃取效率。

**图 5:**
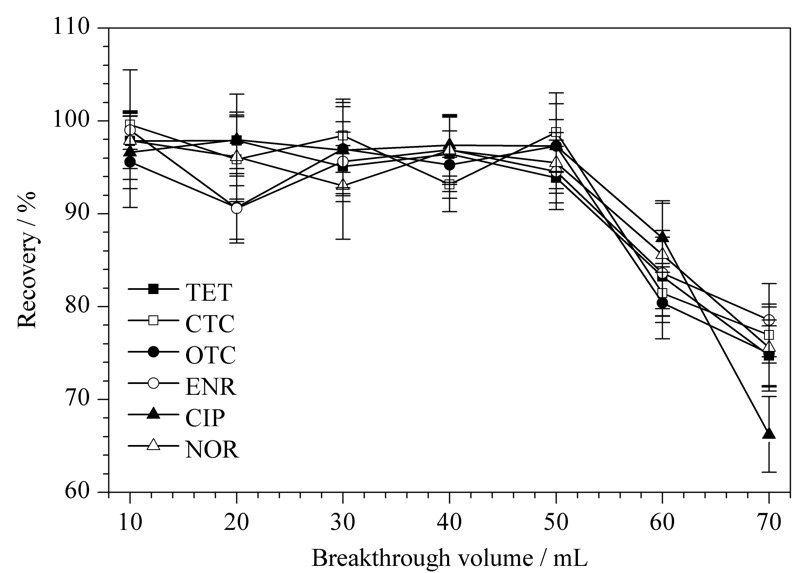
突破体积对6种目标化合物萃取效率的影响(*n*=6)

### 2.4 方法学验证

2.4.1 线性范围、检出限和定量限

各目标物的LOD和LOQ分别以3倍和10倍信噪比(*S/N*)时的加标水平计。结果表明,6种目标分析物的LOD和LOQ分别为0.3~1.5 μg/kg和1.0~5.0 μg/kg, LOD低于国家标准^[12,13]^ (氟喹诺酮类LOD: 20 μg/kg,四环素类LOD: 50 μg/kg)和行业标准^[11]^(四环素类LOD: 50~100 μg/kg),提示本文方法检测灵敏度更优。

空白样品按1.3节进行前处理,得到空白样品溶液,加入不同体积的混合标准溶液,得到系列浓度的基质匹配工作溶液,按1.4节进行UPLC-MS/MS分析。以测得的目标物峰面积为纵坐标、对应的加标水平为横坐标,分别绘制标准曲线。各目标物标准曲线的线性范围为其各自的LOQ~1000 μg/kg(约为所有目标物MRL的1.5~10倍),线性关系良好,决定系数(*R*^2^)均大于0.999(见表2)。

**表 2:** 6种目标物的线性范围、线性方程、*R*^2^、LOD和LOQ

Compound	Linear range/ (μg/kg)	Linear equation	R^2^	LOD/ (μg/kg)	LOQ/ (μg/kg)
TET	2-1000	y=127.0x+128.2	0.9991	0.5	2.0
CTC	5-1000	y=120.0x+102.8	0.9999	1.5	5.0
OTC	5-1000	y=208.5x+29.4	0.9993	1.5	5.0
ENR	5-1000	y=201.2x+48.6	0.9997	1.0	5.0
CIP	2-1000	y=144.5x+48.0	0.9995	0.7	2.0
NOR	1-1000	y=176.8x+25.7	0.9998	0.3	1.0

*y*: peak area; *x*: spiked level, μg/kg.

2.4.2 准确度和精密度

为了评估方法的准确度和精密度,在空白样品中加入不同体积的混合标准溶液,分别得到低、中、高(LOQ、200和1000 μg/kg)3个加标水平的模拟样品,每个加标水平制备6个平行样(*n*=6)。各加标模拟样品按1.3节和1.4节进行前处理和分析。同时以1 d内和连续4 d低、中、高3个加标水平下各目标物含量的相对标准偏差(RSD)计日内和日间精密度。结果如表3所示,各目标物的相对回收率为94.37~102.82%,日内及日间RSD均小于10%。本文方法准确度结果与国家标准^[12,13]^和行业标准^[11]^相比更优,精密度结果相当。

**表 3:** 淡水鱼中6种目标物的回收率和RSD

Compound	Spiked level/(μg/kg)	Intra-day (n=6)/%			Inter-day (n=4)/%	
		Recovery±SD	RSD		Recovery±SD	RSD
TET	2	102.13±6.17	6.04		96.58±8.79	9.10
	200	101.51±4.72	4.65		98.40±5.18	5.26
	1000	99.77±2.37	2.38		100.72±4.49	4.46
CTC	5	98.69±7.84	7.94		101.93±8.16	8.01
	200	98.94±6.23	6.30		97.48±5.76	5.91
	1000	101.24±5.97	5.90		98.34±4.76	4.84
OTC	5	94.37±7.61	8.06		97.65±7.37	7.55
	200	95.81±4.92	5.14		98.46±5.91	6.00
	1000	98.36±3.52	3.58		99.04±4.06	4.10
ENR	5	101.63±7.55	7.43		102.68±7.96	7.75
	200	98.23±7.43	7.56		94.99±6.08	6.40
	1000	99.58±4.66	4.68		96.02±4.97	5.18
CIP	2	101.37±6.88	6.79		95.27±7.93	8.32
	200	97.28±4.96	5.10		96.04±5.12	5.33
	1000	99.79±4.79	4.80		97.55±4.28	4.39
NOR	1	102.82±6.50	6.32		95.07±8.35	8.78
	200	98.77±4.35	4.40		98.13±6.21	6.33
	1000	100.43±3.96	3.94		100.24±4.96	4.95

2.4.3 基质效应

为了评价PDA-PS NFsM材料对鱼肉基质的净化能力,比较了固相萃取前后加标淡水鱼样品中各目标物的提取离子流色谱图(见图6)。可见,在未经PDA-PS NFs SPE小柱净化浓缩前,色谱图中可见杂质峰存在,且响应值较低;经过固相萃取后,杂质峰明显降低,目标物响应值明显升高,显示了PDA-PS NFsM材料良好的基质净化能力和目标物富集能力。

**图 6:**
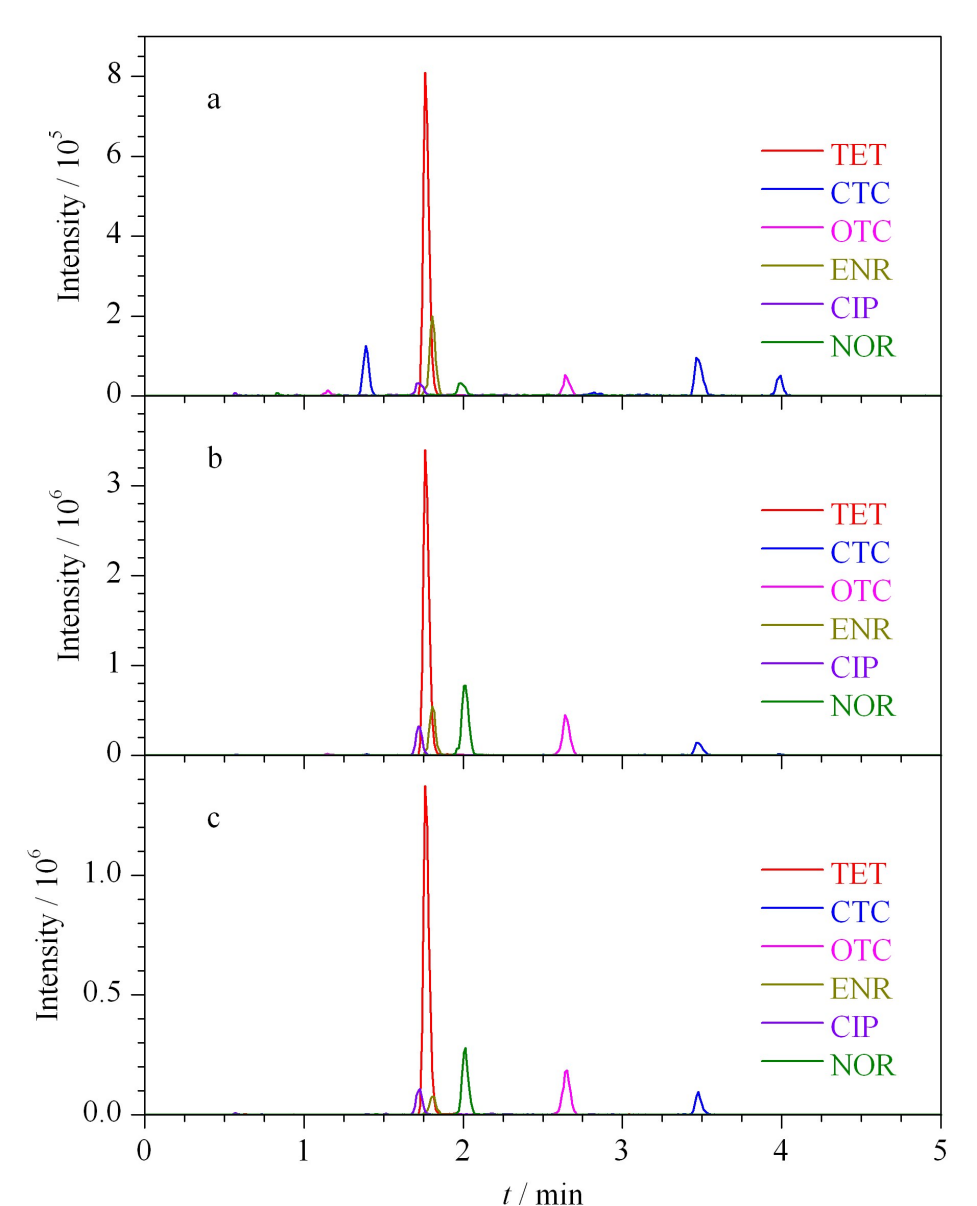
6种目标物的提取离子流色谱图

基质效应=(基质匹配工作溶液中各目标物的峰面积-标准工作溶液中各目标物的峰面积)/标准工作溶液中各目标物的峰面积。空白样品经提取和固相萃取,可得加标(200 μg/L)基质匹配工作溶液,将其与同浓度的标准工作溶液分别行UPLC-MS/MS分析,计算固相萃取后的基质效应。同理对空白样品进行提取,但不固相萃取,可计算得到固相萃取前的基质效应。

结果表明,固相萃取前的基质效应为-12.98%~-38.68%,而在基于PDA-PS NFsM的固相萃取后,每个目标分析物的基质效应显著降低至-2.15%~-7.36%,表明基于PDA-PS NFsM的固相萃取有效净化了样品基质。

### 2.5 与其他方法比较

将本方法与文献^[39,40,41,42]^方法进行了比较(文献检索策略:关键词为水产品、四环素类或氟喹诺酮类药物残留、固相萃取和质谱检测;检索年份为2010~2020;检索数据库为知网、Web of Science)。结果如表4所示,基于PDA-PS NFsM材料的优越性能,本文吸附剂PDA-PS NFsM用量只及文献方法的4%~40%。各目标物的工作曲线线性范围更宽,LOD相当,回收率更高而RSD较低,表明本文方法具有良好的灵敏度、准确度和精密度,具有较好的实际应用潜力。

**表 4:** 本方法与其他方法的比较

Detection method	Adsorbents	Linear range/ (μg/kg)		LOD/ (μg/kg)	Recovery/%		RSD/%		Ref.
LC-MS/MS	C18 (50 mg)	-		0.1-10	70-	120	2.56-	17.18	[39]
UPLC-MS/MS	C18 (100 mg)	5-	100	0.1-10	80.1-	124.8	0.87-	20.09	[40]
UPLC-MS/MS	PSA (200 mg) and C18 (50 mg)	0.2-	200	0.1-1.6	81.6-	96.6	3.6-	9.2	[41]
LC-MS/MS	C18 (500 mg)	5-	50	0.5-5.0	47-	99	4-	17	[42]
UPLC-MS/MS	PDA-PS NFsM (20 mg)	1-	1000	0.3-1.5	94.37-	102.82	2.38-	9.10	this method

### 2.6 实际样品检测

16份淡水鱼样品按1.3节和1.4节进行前处理和分析,每份样品做3个平行。结果表明,3份样品中检出了药物残留,其中1份样品检测到ENR和CIP,残留量分别为60.22 μg/kg和31.90 μg/kg,另2份样品中检出了TET,残留量分别为19.41 μg/kg和26.32 μg/kg。采用国家标准^[12,13]^对16份样品进行了检测,得到的检出情况及测得水平与上述结果一致,表明了本文方法实际应用可行。

## 3 结论

本文将PDA与NFsM的优势相结合,研制了PDA-PS NFsM材料作为SPE介质,PDA-PS NFsM材料不仅可同时提取四环素类和氟喹诺酮类药物残留,且对复杂的样品基质具有良好的净化能力。建立了基于PDA-PS NFsM材料的SPE技术,结合UPLC-MS/MS,研发了同时测定淡水鱼中四环素、金霉素、土霉素、恩诺沙星、环丙沙星、诺氟沙星的检测新方法。方法学考察的结果表明,本文方法灵敏度、准确度和精密度与国家标准、行业标准或文献方法相当或更优,能满足实际样品检测的要求。
